# The Unpredictable Ulnar Nerve—Ulnar Nerve Entrapment from Anatomical, Pathophysiological, and Biopsychosocial Aspects

**DOI:** 10.3390/diagnostics14050489

**Published:** 2024-02-24

**Authors:** Erika Nyman, Lars B. Dahlin

**Affiliations:** 1 Department of Biomedical and Clinical Sciences, Linköping University, 581 85 Linköping, Sweden; lars.dahlin@med.lu.se; 2 Department of Hand Surgery, Plastic Surgery and Burns, Linköping University Hospital, 581 85 Linköping, Sweden; 3 Department of Hand Surgery, Skåne University Hospital, 205 02 Malmö, Sweden; 4 Department of Translational Medicine—Hand Surgery, Lund University, 205 02 Malmö, Sweden

**Keywords:** ulnar nerve, ulnar nerve entrapment, cubital tunnel syndrome, simple ulnar nerve decompression, ulnar nerve transposition, revision surgery, pain

## Abstract

Peripheral nerves consist of delicate structures, including a rich microvascular system, that protect and nourish axons and associated Schwann cells. Nerves are sensitive to internal and external trauma, such as compression and stretching. Ulnar nerve entrapment, the second most prevalent nerve entrapment disorder after carpal tunnel syndrome, appears frequently at the elbow. Although often idiopathic, known risk factors, including obesity, smoking, diabetes, and vibration exposure, occur. It exists in all adult ages (mean age 40–50 years), but seldom affects individuals in their adolescence or younger. The patient population is heterogeneous with great co-morbidity, including other nerve entrapment disorders. Typical early symptoms are paresthesia and numbness in the ulnar fingers, followed by decreased sensory function and muscle weakness. Pre- and postoperative neuropathic pain is relatively common, independent of other symptom severity, with a risk for serious consequences. A multimodal treatment strategy is necessary. Mild to moderate symptoms are usually treated conservatively, while surgery is an option when conservative treatment fails or in severe cases. The decision to perform surgery might be difficult, and the outcome is unpredictable with the risk of complications. There is no consensus on the choice of surgical method, but simple decompression is relatively effective with a lower complication rate than transposition.

## 1. Introduction

The ulnar nerve is one of the major nerve trunks that innervate the upper limb. It has a vulnerable anatomical location, particularly at the elbow, where it is superficially located and may be subjected to “internal entrapment” or “external” compression and stretching during elbow flexion. Nerve entrapment affecting the ulnar nerve—ulnar nerve entrapment—can be challenging to treat. The symptoms that affected patients present, often with a prominent pain component, may be difficult to judge, and even defining a precise diagnosis of ulnar nerve entrapment is demanding due to the heterogeneities of the patient population, sometimes extensive comorbidity, and influencing socioeconomic factors [1,2,3,4,5,6,7,8]. There are speculations that the ulnar nerve and its large, myelinated nerve fibers are more susceptible to diseases and injuries because of the limited “reserve capacity” of nerve fibers [9]. While there are many studies on ulnar nerve entrapment, high-quality prospective, randomized, and controlled studies are rare and often complicated by problems, such as grading of symptoms, agreement on surgical indications, and lack of standardized follow-up in sometimes heterogenic cohorts. This concise review focuses on the issue of ulnar nerve entrapment at the elbow, highlighting its pathophysiological and biopsychosocial aspects [10]. It emphasizes the unpredictable nature of this condition both before and after surgery, where pain can often be a prominent symptom. There is no consensus on the required diagnostic methods, indications for surgery, or selection of surgical procedures depending on stages of the disorder or severity of the symptoms in the international literature, which is why the present review mainly reflects the opinion of our geographical healthcare areas. 

## 2. The Peripheral Nerve

**Figure 1:**
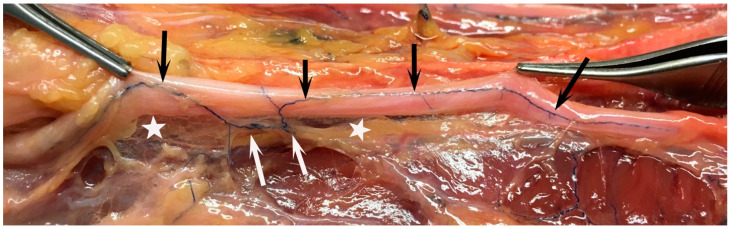
Dissection of a cadaver intraarterially perfused with a rubber-ink substance to visualize the blood vessels in an ulnar nerve. The photo shows the ulnar nerve lifted and exposed. Note the segmentally approaching blood vessels (white arrows) with a coiled appearance (see right white arrow), which are approaching the nerve in the mesoneurium (thin visible tissue; marked by stars). The result is an intraneural, axially running intraneural blood vessel system (black arrows).

Peripheral nerve trunks are highly sensitive and well-vascularized structures. Blood vessels nourish the nerve trunks as they approach the nerve through a thin membrane called the mesoneurium. This is similar to the mesenterium that supports the intestines. The segmentally approaching blood vessels have a coiled appearance that compensates for the excursion of the nerve during movement (Figure 1), which is particularly important for the ulnar nerve at the elbow level during flexion and extension movements. The intraneural blood vessels are divided into plexa in the various parts of the nerve. The nerve trunks consist of bundles of nerve fibers surrounded by a protective membrane called the perineurium, which forms a fascicle. The intrafascicular content, known as the endoneurium, consists of the nerve fibers and the intrafascicular blood vessels of different sizes, mainly capillaries, macrophages, fibroblasts, mast cells, and collagen [11]. The different nerve trunks in the extremities contain a varying number of fascicles, which are bound by a loose connective tissue called the epineurium [12]. The amount of connective tissue in the three different components mentioned varies concerning nerve protection, depending on their location in the extremity and along the nerve. Superficial nerves have more connective tissue, and the nerve contains more connective tissue around joints [11].

The intraneural blood vessels are easily affected by compression, which can cause the blood vessels in the epineurium to become more permeable, resulting in edema [13,14]. The blood vessels approaching the endoneurial space, thus creating a particular endoneurial capillary network, go obliquely through the perineurium [11] (Figure 1). The epineurial blood vessels are more sensitive to trauma, while the endoneurial capillaries are more resistant [13]. If a fascicle experiences trauma and develops edema through increased permeability of the endoneurial blood vessels, the perineurium prevents the edema from draining, leading to compartment syndrome in miniature [15].

## 3. The Ulnar Nerve

### 3.1. Anatomical Reflections

**Figure 2:**
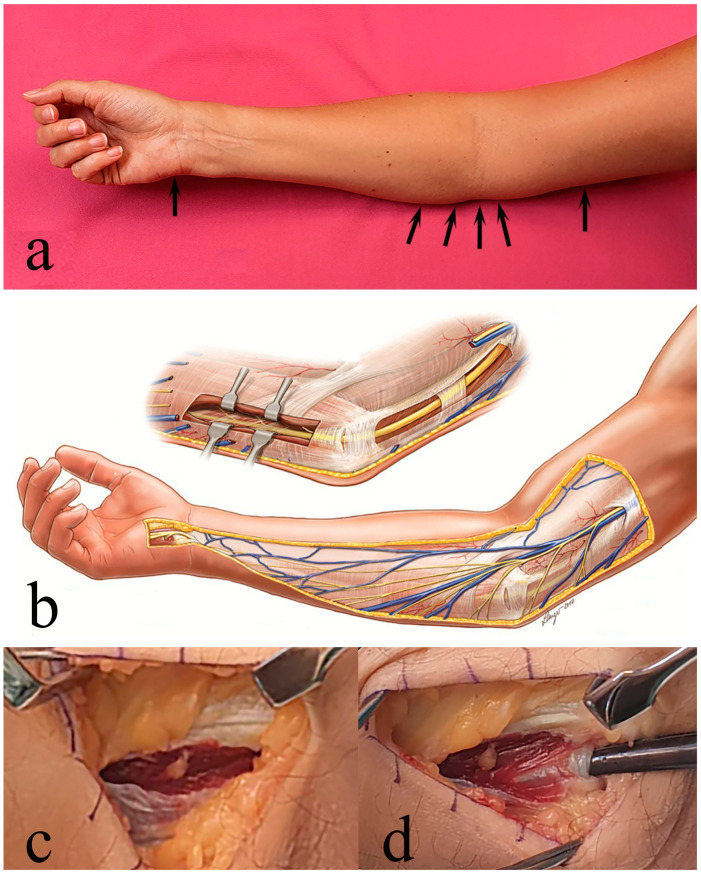
Photos and schematic image by potential site for compression (arrows in (**a**)). See text for details. The anatomy of the ulnar nerve with depicted entrapment sites is visualized in (**b**). The probable most common entrapment site is at the ligament of Osborne, being the proximal edge of the fascia extending between the two heads of the flexor carpi ulnaris muscle (below the forceps in (**d**) after division of the superficial aponeurosis in (**c**)). The schematic drawing (**b**) was published, after light modification, with kind permission from hand surgeon Martin Langer, Münster, Germany.

The ulnar nerve can be compressed at various locations, including the elbow and wrist, and may also be affected by external factors due to its anatomical location. Anatomical variations, such as Martin-Gruber and Riche-Cannieu anastomosis [16], can also make diagnosis difficult. The nerve is formed by the C8 and Th1 spinal nerve roots, which create the inferior trunk of the brachial plexus, followed by the medial cord. The inferior trunk is located near the upper part of the thoracic space and pulmonary tissue, which means that tumors in the lung, or in the lower part of the brachial plexus [17], can affect the inferior trunk and cause symptoms in the ulnar nerve’s innervation area. The nerve travels down the medial side of the humerus, passing the arcade of Struthers, the retro-epicondylar groove, and entering the cubital tunnel. The roof of the cubital tunnel is called the cubital tunnel retinaculum. Subsequently, the ulnar nerve passes beneath the aponeurosis of the flexor carpi ulnaris muscle and its two origins, where the upper edge of the aponeurosis forms the ligament of Osborne, which is probably to the most frequently affected site in ulnar nerve entrapment [18] (Figure 2).

The ulnar nerve is in the forearm, located beneath the flexor carpi ulnaris muscle, which it innervates along with the ulnar half of the flexor digitorum profundus muscle. It then travels with the ulnar artery down to the canal of Guyon. The nerve splits into three branches distally: a dorsal sensory branch, a volar superficial sensory branch (often early branching in a proper and common digital nerve), and a deep motor branch. The deep motor branch goes down into the deep part of the hand beneath the edge of an aponeurosis around the hamulus of the hamate bone and innervates most of the hypothenar and intrinsic muscles. The dorsal sensory branch leaves the main trunk around 5–8 cm proximal to the canal of Guyon and innervates the dorsal ulnar part of the hand. This is important clinical knowledge for diagnosing the level of nerve entrapment. Interestingly, the fascicles containing the volar sensory and motor nerve fibers to the fingers and hand are located more peripherally in the nerve at the elbow level and are more sensitive to trauma [12,19].

### 3.2. Pathophysiological Considerations

When treating nerve entrapment disorders, it is important to consider the underlying pathophysiological mechanisms. If the patient experiences intermittent symptoms that rapidly disappear, such as during the extension of the elbow from flexion, the cause is likely due to the involvement of the intraneural microvessels, possibly in combination with a slight edema [13]. During flexion, the pressure below the ligament of Osborne increases significantly, exceeding the intraneural perfusion pressure [14,20]. If the patient experiences continuous symptoms, there are likely more structural changes in the nerve, including myelin damage and axonal degeneration, which can be detected through electrophysiological examination [21,22,23,24,25,26]. Severe axonal degeneration can lead to muscle atrophy, which is a poor prognostic sign and may require further surgical intervention. It is also important to consider an underlying neuropathy in the nerve, such as diabetic neuropathy, which can make the nerve more susceptible to compression, as seen in the well-studied case of carpal tunnel syndrome [23,27,28,29,30].

The concept of double crush was introduced to explain the increased sensitivity of nerves in nerve entrapment disorders, including the concept of “sick” neurons with an underlaying neuropathy as in diabetes [27]. In this concept, a nerve, such as the ulnar nerve, may be more sensitive to compression at a distal level if it is also affected more proximally, such as at the spinal nerve root level [27]. Additionally, a reversed double crush may occur, where the nerve can be more sensitive closer to its origin if it is affected further down its pathway [31].

## 4. Ulnar Nerve Entrapment

### 4.1. Potential Anatomic Entrapment Sites

The ulnar nerve can be compressed and affected at various points around the elbow, such as the arcade of Struthers, the medial intermuscular septa, at the level of the medial epicondyle at the ulnar groove, or under the ligament of Osborne, the latter typically the most crucial site—the cubital tunnel, as well as in the forearm where it passes under the flexor carpi ulnaris muscle [32,33,34] (Figure 2). It is important to note that the ulnar nerve may be severely affected due to different types of traumas to the elbow region, including fractures and subsequent closed or open repositioning with various osteosynthesis procedures, including heterotopic ossification around the elbow. In trauma cases, it may be necessary to mobilize and transpose the nerve during surgery for protection. Anatomical structures, such as accessory muscles (i.e., most often reported by an anconeus-epitrochlearis muscle or a displaced triceps muscle belly), as well as inflammatory joint conditions (i.e., rheumatoid arthritis, osteoarthritis in the joint, or extraneural and intraneural cysts originating from the elbow joint), should also be considered as etiologies to the ulnar nerve entrapment at the elbow. Other soft tissue masses, such as lipomas, fibrolipomatous hamartoma, giant cell tumors or tophaceous gout, may be an underlaying cause. These causes of the ulnar nerve entrapment should be handled as a part of the entrapment problem (Table 1).

### 4.2. Patients, Symptoms, and Clinical Findings

Ulnar nerve entrapment may affect patients of all ages, but it is most common in those 40–50 years old. The risk of being affected is relatively equal between sexes, eventually with a slight overweight for women. Risk factors are, for example, obesity, smoking, diabetes, working with vibrating tools, and manual work with repetitive work tasks [35,36,37]. Dysregulation of blood lipids is also relevant for the development of nerve entrapment disorders, such as ulnar nerve entrapment, and neuropathies [38], as well as for the recovery after a nerve injury requiring nerve regeneration, like a severe case of ulnar nerve entrapment [39]. Carpal tunnel syndrome is often diagnosed at the same time, and problems from the neck or shoulders may be over-representative [40,41] (Table 1).

Patients with ulnar nerve entrapment at the elbow usually present with paresthesia and numbness in the ulnar fingers, including the dorsal ulnar part of the hand. This is often accompanied by nocturnal problems, especially when the elbow is flexed during sleep or when the pressure against the nerve is increased. Additionally, patients may experience discomfort around the elbow and forearm, including medial pain. Questions about the pain, such as severity, occurrence during the night and day, and frequency, are important to ask [42,43]. In addition, a typical response to the following questions would be “yes”: Do you feel any numbness or tingling in your ring or little finger while talking on the phone? Do you experience any difficulties while lying in bed, reading a book, or holding an electronic device? These questions are reflected in the questionnaire PRUNE [42,43].

**Figure 3:**
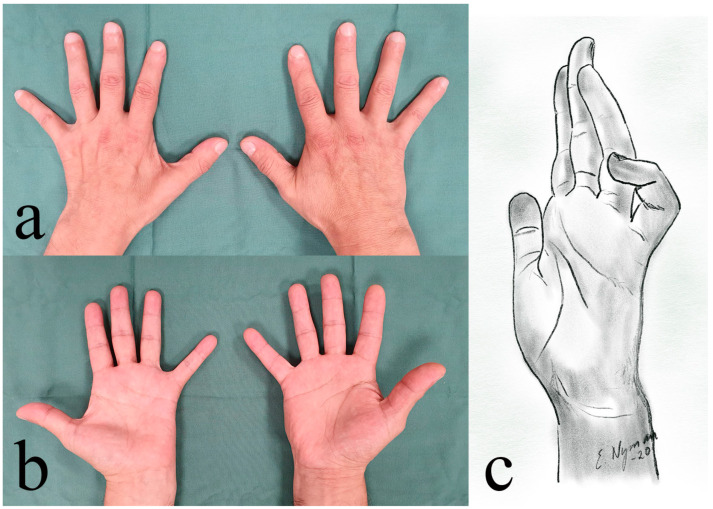
Patient with severe ulnar nerve affection (left hand; note atrophy of the intrinsic muscle and abducted little finger due to imbalance between the abductor digit minimi and the third volar interosseous muscles) with a dorsal (**a**) and volar (**b**) view as well as a schematic drawing (**c**) with severe clawing.

**Figure 4:**
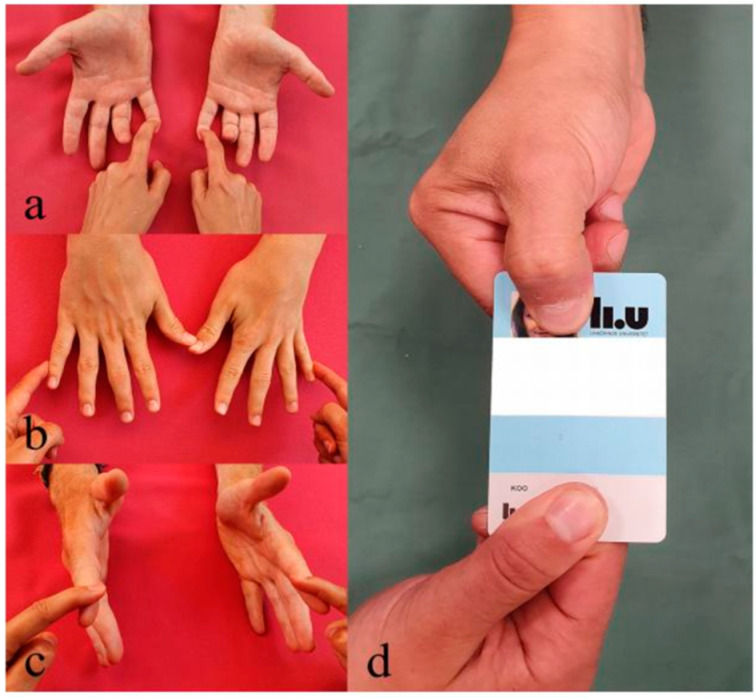
Technique for examining the motor function of the ulnar nerve, where function in the flexor digitorum profundus muscle to the little finger (**a**), abductor digiti minimi muscle (**b**), and first dorsal interosseous muscle (**c**) is examined, as well as the dysfunction of the adductor pollicis muscle visualized with Froment’s sign ((**d**); compensation through more flexion of the first interphalangeal-joint of the thumb; upper hand).

As the patient’s condition progresses, they may experience motor symptoms, such as impaired dexterity and weakness in the hand, along with a constant decrease in sensory function. Patients may experience a positive Tinel’s sign, discomfort when touching the skin over the ulnar nerve, and other objective findings, such as weakness in the flexor digitorum profundus muscle to the little and ring fingers, hypothenar muscles, and interosseous muscles, most prominently observed in the first dorsal interosseous muscle (Figure 3 and Figure 4).

When the nerve is severely affected, inducing axonal degeneration, a marked decrease in strength is observed, with atrophy of the intrinsic muscles and clawing of the two ulnar fingers (Figure 3). In this condition, a positive Froment’s sign is often observed (Figure 4). Pain that occurs near the elbow and radiates proximally or distally can be a severe symptom that may be experienced by the patient both early and late in ulnar nerve entrapment. There is an old clinical grading of ulnar symptoms and clinical findings according to McGowan [44], which has been modified by Dellon [45,46]. However, a more detailed grading of the symptomatology with a connection to neurobiological mechanisms and different treatment strategies could be more useful in clinical practice.

When diagnosing ulnar nerve entrapment, it is important to consider the sensory and motor functions of the entire length of the nerve—from the neck down to the target—thereby reflecting the nerve entrapment as a “cellular injury” to the neuron along the nerve. The clinical diagnostic should include several different examination parameters, such as tests of sensation and motor function [e.g., tests with monofilaments, two-point discrimination, tests for muscle strength according to the Medical Research Council (MRC) scale in individual ulnar and median nerve innervated muscles], as well as different provocation tests (e.g., flexion of elbow or wrist by the examiner asking the patient about any paresthesia in little and ring fingers; Tinel’s sign at potential affected sites for comparison with the contralateral side) [1]; all with their different specificity and sensitivity. It should be noted that no single test is 100% specific or sensitive for ulnar nerve entrapment [47,48,49]. In addition, the examination should also include tests specifically for median nerve entrapment, which often occurs in carpal tunnel syndrome, radial nerve entrapment, and other tests if the surgeon suspects spinal nerve root affection in the neck. Evaluation of any comorbidities possibly affecting the risk for development of the disorder and prognosis of treatment, such as diabetes, vibration exposure, polyneuropathy, as well as work-related risk factors, should be considered [50,51]. More importantly, it is essential to consider differential diagnosis, as the nerve could be affected by different conditions along its course from the spinal cord down to the target.

### 4.3. Discussion about Consensus of Diagnostic Criteria and Diagnosis of Ulnar Nerve Entrapment

Generally, there is no international consensus on the diagnosis of ulnar nerve entrapment since the specific diagnostic criteria may vary with national guidelines and traditions, which also includes the indication for surgery and selection of surgical method. Recently, a US-based study using the Delphi method reported a consensus concerning potential diagnostic criteria for ulnar nerve entrapment despite study limitations [52]. The authors presented six criteria for diagnosis: (a) paresthesia in ulnar nerve distribution, (b) symptoms increased by elbow flexion and positive elbow flexion test; (c) positive Tinel sign at elbow; (d) atrophy/weakness/late findings (e.g., claw fingers of the ring and small finger and Wartenberg or Froment signs) of ulnar nerve innervated hand muscles; (e) loss of two-point discrimination in the ulnar nerve innervation area; and (f) similar ipsilateral symptoms after successful treatment on the contralateral side [52]. One may argue against such criteria since it involves several criteria related to severity and does not include more sensitive tests for sensation, such as the Semmes-Weinstein monofilament test. In the discussion about diagnostic criteria, many authors highlight the lack of a standard algorithm for diagnosis as well as for treatment [53], where other evaluation methods also should be considered or recommended (see below *Complementary investigations in ulnar nerve entrapment*). The balance between using the patient’s history, the clinical findings, and results from any additional diagnostic procedures to set the diagnosis of ulnar nerve entrapment may vary between geographical areas from an international perspective. The type of referred cases that the individual treating surgeon judges at their outpatient clinic may also have an impact on diagnostic criteria and the selection of any surgical technique. The selection of any procedure may not only be influenced by the surgeon but also by patient demographics [54]. The lack of diagnostic criteria with a related classification based on the severity of symptoms is obvious and should be a matter of future research.

### 4.4. Socioeconomic Aspects

Generally, socioeconomic position, measured by education, income, occupational status, and wealth, is strongly connected to mortality and low income, but not to low education or race. The socioeconomic factor remains predictive for mortality even when adjusting for major health risk behaviors [55,56,57]. Socioeconomic factors are also well known to affect the patient’s health and the outcome of orthopedic surgery in general [58] and ulnar nerve entrapment in particular [6,7]. A low educational level has specifically been shown to be a risk factor for ulnar nerve entrapment [59]. In a national registry study on surgically treated primary cases of ulnar nerve entrapment, these patients had lower levels of education, higher social assistance dependence, a high proportion of unemployment, and lower earnings compared to the general population [7] (Table 1). In the same study, manual labor was not related to having surgery for ulnar nerve entrapment nor affected surgical outcome [7]. However, in other studies, manual labor has been pointed out as a risk factor for developing ulnar nerve entrapment [59,60,61], which may be based on the definition of manual work. In other studies, “economic distress” was not associated with the self-reported duration of symptoms or the severity of the ulnar nerve entrapment [62]. Differences may be related to the geographical areas across the Western world and the influence on the social insurance systems in the specific countries. Further on, women with ulnar nerve entrapment are more socioeconomically deprived than men and rate their disabilities greater than men do both before and after surgery. Interestingly, they also improve more with surgery [6,63]. In contrast, male sex and older age have been reported to be associated with a more severe ulnar nerve entrapment at presentation at the outpatient clinic [23,62]. A further aspect is that sex differences in pain sensitivity are complex. Women have a lower pain threshold and experience greater temporal summation of pain to repeated or dynamic stimuli than men, while they have a greater ability to adapt to sustained stimuli as well as habituation to repeated stimuli, which may be based on two suggested mechanisms: the “sensing response” and a “modulating/coping response” [64]. The socioeconomic factors are of outmost relevance since biopsychosocial aspects should always be considered when taking care of patients, especially those with ulnar nerve entrapment. Further research is needed to clarify the impact of these aspects for the diagnosis, indication for surgery, and outcome of surgery. 

### 4.5. The Painful Ulnar Nerve

Neuropathic pain can occur in connection with a variety of nerve injuries, including compressed nerve trunks and nerves affected by an underlying neuropathy, where the complaints of the patients consist of ongoing or intermittent spontaneous pain characterized by burning, pricking, or squeezing qualities that are easily evoked by light touch or cold exposure to the area of the affected nerve [65,66,67]. Spontaneous ectopic activity from the injured nerve, which may be particularly problematic in partial nerve injuries, may be an underlaying mechanism. In addition, it is not uncommon that the evoked pain includes stimulation of and pain in neighboring areas innervated by other nerve trunks [65,68]. Further, maladaptive mechanisms occur based on the peripheral and central sensitization of the nociceptive pathways, with aberrant processing inducing allodynia [67]. Pain is not only a stimulus transmitted from the nociceptors and through the ascending and descending tracts but is also cortically processed to finally give the patient an experience of pain [69]. The present research highlights how several different pharmacological substances used to treat pain exert their action, including the commonly used gabapentin and pregabalin [67]. It is mandatory to evaluate the pain and its different modality carefully and meticulously in relation to sensory function in patients with nerve injuries, including those with pain in relation to an ulnar nerve entrapment, independently of whether primary or revision surgery is performed or not [70]. Pain seems to be a prominent feature among the patients with persistent or recurrent ulnar nerve entrapment (meta-analysis; pain 82%, sensory dysfunction 81%, and motor dysfunction 53%) [71]. Evaluation of pain may include different pain assessment tools, such as Numerical Rating Scale (NRS), pain Visual Analog Scale (VAS), McGill´s Pain Questionnaire (MPQ) and short form MPQ, Pain Disability Index (PDI), Cold Intolerance Symptom Severity (CISS) Scale, Patient-Reported Outcome Measurement Information System (PROMIS) with subsets Pain Intensity, Pain Interference, Pain Behavior, and Neuropathic Pain Quality Scale, as well as the questionnaires Douleur Neuropathique 4 (DN4) and HQ-8 [70,72,73]. Pain should also be related to the outcome of sensory function using a variety of assessment tools, such as the Semmes-Weinstein monofilament test, two-point discrimination, pick-up test, ten test, shape texture identification test (STI), manual tactile test, and thermal sensitivity test [70]. Chronic severe neuropathic pain, for example, associated with ulnar nerve entrapment, affects almost all aspects of daily life, including the impact on life satisfaction, overall health status, mood and emotions, struggle with self-image, changes in life roles, and sexual life [74,75]. Sleeping disturbances are well-known in patients with chronic neuropathic pain, including patients with neuroma, as well as in patients with ulnar nerve entrapment and carpal tunnel syndrome [76,77,78]. Regaining the ability to sleep has been emphasized as the turning point to getting their lives back among patients with chronic pain in conjunction with surgery for ulnar nerve entrapment [74,78,79] Further on, the invisibility of pain is pointed out as frustrating and raising the emotion of not being believed [74].

Pain, being present preoperatively or in conjunction with primary and revision surgery for ulnar nerve entrapment, is a symptom not particularly well highlighted or structurally evaluated in the literature, despite included questions about pain in evaluation studies of ulnar nerve entrapment [42,73,80,81]. Still, pain of different modalities is the most prevalent symptom in many patients [71]. Interestingly, medial elbow pain can also exist independently from the ulnar nerve entrapment, indicating that medial elbow pain is not a clear diagnostic symptom of ulnar nerve entrapment [81]. The more extensive surgical procedures, like any type of transposition, are associated with complications, such as paresthesia and elbow pain, as well as a risk for revision surgery [2,82,83]. Pain can be reduced by revision surgery, particularly using a submuscular, but sometimes also a subcutaneous, ulnar nerve transposition, for ulnar nerve entrapment, but the application of strict principles in primary surgery for the condition is even more important [84]. 

At a tertiary referral center treating patients with ulnar nerve entrapment, emergent neuropathic pain was the most frequent postoperative symptom [40]. Neuropathic pain in conjunction with surgery for ulnar nerve entrapment is a challenging issue, as all types of pain are related to nerve injuries [85]. A variety of systemic pharmacological substances have been suggested as a tool to treat the pain, including paracetamol, in combination with a selective COX-2 inhibitor, opioids, gabapentin, pregabalin, duloxetine, venlafaxine, and amitriptyline [65,67,85]. However, opioids are not recommended due to the risk of overuse, and overuse of opioids and gabapentinoid drugs, as reported after surgery for ulnar nerve entrapment alone, or in combination with carpal tunnel syndrome, is also higher than for carpal tunnel syndrome alone [86]. Locally, lidocaine-medicated patches, through their action of blockade of voltage-gated sodium channels and stabilization of the neuronal membranes as well as inhibition of ectopic discharge, are applicable to treat peripheral pain, e.g., around a surgical scar around the elbow [65]. Similarly, single, or repeated application of capsaicin 8% patches at areas with intense allodynia, thereby reducing the intraepidermal nerve fiber density (IENFD), is also an efficient alternative, which can be repeated every third month [65]. The exact mechanism(s) to locally reduce pain by subcutaneously or intradermally applying botulinum toxin A is not known, but the treatment may be efficient [65,67]. These treatment modalities can be utilized by the treating surgeon, but the use of physiotherapy seems to have a limited place in pain treatment connected to surgery for ulnar nerve entrapment [87]. Other treatment strategies involve graded motor imagery and mirror therapy, however, mostly used in the treatment of problems related to amputations [85]. Transcutaneous electrical nerve stimulation (TENS) is a suitable adjunct to pharmacological intervention, which has been used for a long time despite weak scientific ground [88,89]. Whether transcranial magnetic stimulation (TMS) has a place in the treatment of neuropathic pain and pain related to ulnar nerve entrapment, in particular, remains to be proven [85]. As a complement to medication and other treatment modalities, cognitive behavioral therapy with the support of a psychologist might be needed [90]. 

Finally, pulsed radiofrequency stimulation (PRF) has been applied in the strategy of pain treatment, including neuralgia, where an electrical field and heat bursts to the targeted nerves are delivered through the needle tip of a catheter [91], but the technique is only described in a limited number of cases suffering from pain after ulnar nerve entrapment [92]. “Cryoneurolysis” may be a further option to treat localized peripheral pain, for example, related to a nerve branch, in patients after surgery for ulnar nerve entrapment [93]. Spinal cord stimulation (SCS) is one of the neuromodulators that is used to treat neuropathic pain [94,95], which is also an option to treat refractory pain, in combination with all the other techniques, in ulnar nerve entrapment. A combination of a variety of pharmacological possibilities and other methods can be used to determine the optimal treatment for the individual patient. A sign of impaired psychological health in surgically treated patients with ulnar nerve entrapment alone, or in combination with carpal tunnel syndrome, is a reported long-term use of psychotropic drugs among such patients [96]. Thus, it is of outmost relevance to consider all aspects—with a holistic view—in the management of patients with pain treated for ulnar nerve entrapment, where the unpredictable ulnar nerve may elicit severe pain. Therefore, aspects “beyond surgery” should be considered with a multimodal treatment strategy [90,97].

### 4.6. Complementary Investigations in Ulnar Nerve Entrapment

A comprehensive medical history taken by the surgeon and a detailed physical examination are crucial when it comes to the diagnostic procedure. Depending on the findings, other suitable methods should be used to complement the examination. An electrophysiological examination, using specific criteria, can help to grade the damage to the ulnar nerve [98], with a suggested classification [46], but it cannot differentiate between acute traumatic and chronic non-traumatic ulnar neuropathy at the elbow [99]. Ideally, the evaluation should be carried out with an inching technique, where the nerve is stimulated across the short length of a nerve to identify the precise anatomical site where the nerve is affected [100,101,102]. Signs of axonal degeneration and metabolic conduction block, compared to normal findings or only an impaired nerve conduction velocity, could indicate a poorer outcome of the surgical treatment [23]. If other neuropathy, including motor neuron disease, is suspected, a meticulous clinical examination as well as an electrophysiological examination with electromyography should be performed [103]. Depending on what the surgeon suspects is the cause of the symptoms, a conventional X-ray of the elbow, wrist, and cervical spine, as well as an X-ray of the lungs, can be carried out [104]. In addition, MRI imaging is conducted to visualize not only elbow conditions [105], but more importantly, other intra- and extraneural pathological conditions at the elbow, also extending up to the spinal nerve root level in the cervical spine [41,104,106,107,108,109], where the examination can be supported by diffusion tensor imaging (DTI) and tractography [110]. An MRI examination must be carried out in detail based on the clinical examination, focusing on the exact site of the suspected affection. Ultrasound is nowadays often recommended, and its value increases with the development of the technique, resolution, and competence of the examiner to map out nerve affection at different anatomical sites [104,111,112,113,114,115,116,117] (Table 2).

## 5. Treatment

### 5.1. Conservative or Surgical Treatment

The treatment strategies for nerve entrapment disorders target the symptoms. In cases of mild and intermittent symptoms, conservative treatment is recommended, which includes ergonomic advice and the use of orthosis for the night if the patient tolerates it [3,118]. The treatment should be tried for at least three months. The use of NSAIDs has probably no effect and is not recommended, except as an adjunct in pain treatment, but steroid injections (with lidocaine) have been reported to be beneficial [3]. However, such a treatment does not target any cause of entrapment unless an inflammatory condition is present.

**Figure 5:**
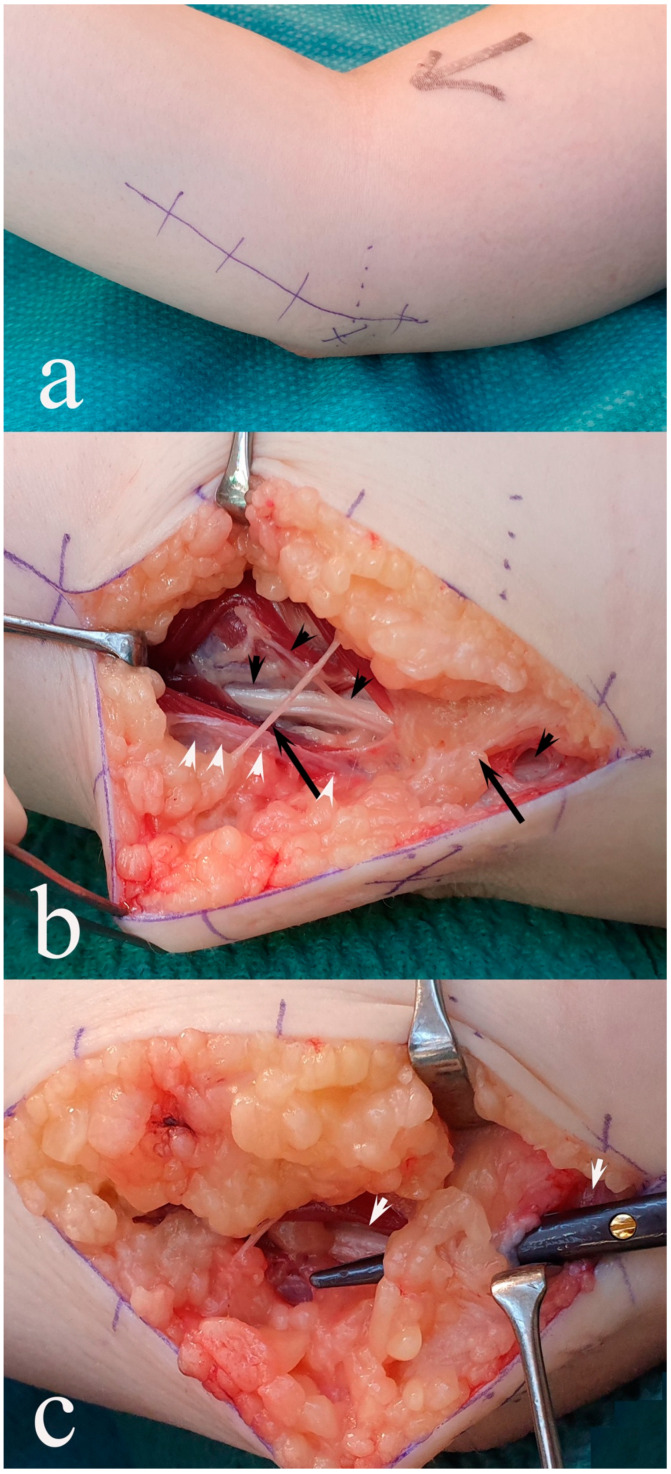
Stages during simple decompression (**a**–**c**) of the ulnar nerve at the elbow. (**a**) Skin incision marked (medial epicondyle marked with a dashed line and ulnar nerve growth marked with a cross); (**b**) ulnar nerve with branches to the flexor carpi ulnaris muscle (black arrowheads) in the depth and spared more superficially located oblique subcutaneous branches (black long arrows). White arrows indicate divided, compressed structures. (**c**) The scissor indicates a slighter non-compressing fascia/fat structure that can be spared (ulnar nerve shown by white arrows).

There is no consensus on the indication for surgical treatment or the choice of surgical method, including the use of preoperative electrophysiology [2,23,82,119,120,121,122,123,124]. There may be several explanations for the lack of consensus, such as the organization of the health care sector, the patient characteristics, the competence of treating surgeon, as well as the local traditions and possible global guidelines. However, if the patient presents with constant and persistent symptoms of different severity, surgery is a clear option. Surgery is typically performed in a bloodless field, either with patients under general anesthesia, an axillary brachial plexus block, or Wide Awake Local Anesthesia No Tourniquet (WALANT). Several surgical techniques can be used, but the ulnar nerve should be treated with care and according to the severity of the condition. In ours and others’ opinions, a simple decompression is the best technique and has a low risk of complications. Direct comparison of various techniques and studies may be problematic since different outcome measurements for evaluation of the surgical procedure have been used [2,125,126,127,128]. Suspected sites of compression are decompressed under loop magnification (Figure 5). 

During the exploration, it is important to be careful with the small, oblique subcutaneous nerve branches. These branches come from the medial antebrachial cutaneous nerve and can usually be found around 1 cm proximally and 3 cm distally to the medial epicondyle. Injuring these branches can lead to significant pain problems. Additionally, there may be one or more motor branches to one of the muscle heads of the flexor carpi ulnaris muscle that can be seen when the ligament of Osborne and fascia of the muscle is divided [18,129] (Figure 2 and Figure 5). 

After a simple decompression, it is typically enough to apply a soft bolstering dressing for two weeks and then proceed with immediate, careful mobilization, being particularly mindful to avoid extensive flexion. A full load is recommended only after 4–6 weeks. After a simple decompression, patients usually return to work at a median time of 6 weeks, independent of the patients having manual or non-manual work [130].

**Figure 6:**
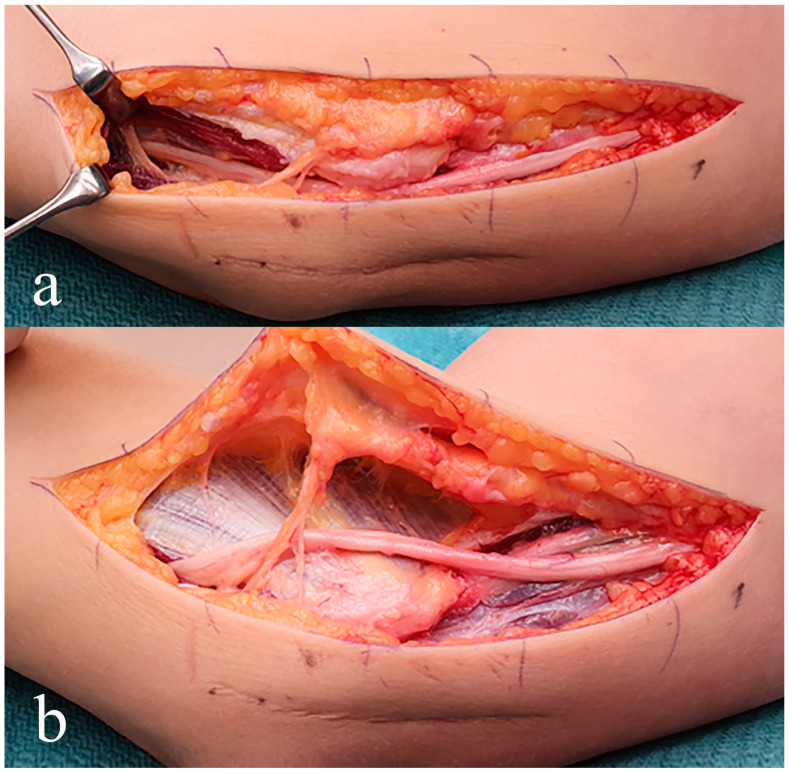
Patient referred to a tertial center for hand surgery due to persistent ulnar nerve symptoms. Note the scar below the incision from the previously performed primary procedure. Subcutaneous ulnar nerve transposition with the skin incision and the nerve mobilized (**a**) and the nerve transposed anteriorly to the medial epicondyle (**b**). During an ulnar nerve transposition, it is crucial to mobilize the nerve extensively, still atraumatic and preserve as many approaching blood vessels as possible, avoiding any bending of the nerve, and resect the lower part of the intermuscular septum, preventing any pressure riding of the nerve across the septum. Resection of the opened aponeurosis of the flexor carpi ulnaris muscle might also sometimes be necessary. After covering the transposed ulnar nerve with subcutaneous tissue, the gliding of the nerve is controlled.

Several other treatment techniques are available for ulnar nerve entrapment [1]. These include medial epicondylectomy, endoscopic nerve decompression, and various nerve transpositions, such as subcutaneous, intramuscular, or submuscular transpositions [2,82,121,122,123,131,132,133]. Again, there is no consensus on the selection of surgical method, a matter that can be influenced by the organization of the health care sector, the individual’s surgeon´s competence with the patient presenting with symptoms of ulnar nerve entrapment, as well as local traditions and possible guidelines. Thus, again, these aspects should be a focus of future research on ulnar nerve entrapment. Ulnar nerve transposition may be recommended only in cases where there is a pre-existing tendency for subluxation in elbow flexion or recurrent ulnar nerve entrapment [121,131,134,135,136,137] (Figure 6). During this procedure, the ulnar nerve is mobilized extensively around the medial epicondyle and repositioned anteriorly. This may require intraneural dissection to spare the nerve branches to the flexor carpi ulnaris muscle and some of the approaching blood vessels to the ulnar nerve must be divided (Figure 1). The nerve can be placed either in the subcutaneous fat (Figure 6) or beneath the muscle fascia (intramuscularly or submuscularly), depending on the technique used. The latter procedure can be carried out with or without bone release from the medial epicondyle. Immobilization in a plaster cast is often used for 3–6 weeks, depending on the procedure, and full load may not be allowed up to 3 months after release of the muscle tissue. The time for return to work after ulnar nerve transpositions is longer than for simple decompression (median time: 8 weeks; odds ratio being similar between subcutaneous and submuscular nerve transpositions) [130]. The most important prognostic factor is the condition of the nerve, specifically the extent of intraneural structural changes, and whether the surgery is as atraumatic as possible. All ulnar nerve surgery, especially ulnar nerve transposition, requires an experienced surgeon. Some authors consider medial epicondylectomy to be less risky than transposition, but complications have been reported [123,138]. Endoscopic nerve decompression is used but usually involves extensive neurolysis distally, which can be questioned [132,139,140,141]. Other techniques, like brief electrical stimulation, have also been reported as favorable as an adjunct to surgery [142]. 

**Figure 7:**
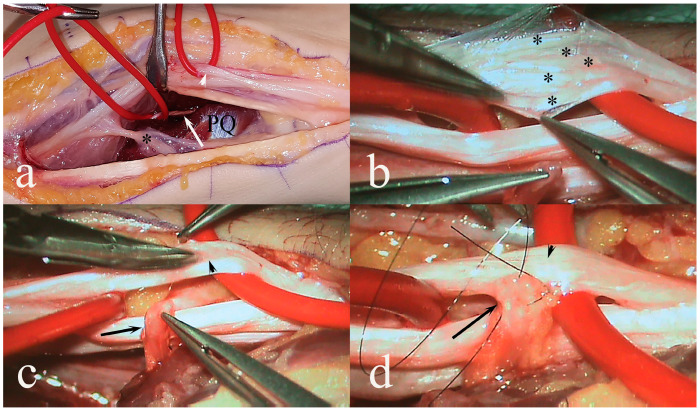
Surgical exploration of the motor ulnar nerve (white arrowhead in (**a**)) distally in the forearm with the dorso-ulnar branch (black asterisk) with the anterior interosseous nerve (AIN; white arrow), explored deep in the forearm just proximally before it enters the pronator quadratus muscle (PQ). AIN is divided just proximally to its first branch in pronator quadratus muscle. The asterisks in (**b**) indicate different fascicles of the motor branch of the ulnar nerve. The AIN is connected end-to-side without any tension (black arrow in (**c**,**d**)) to the motor ulnar branch (black arrowhead) as a nerve transfer as a “supercharge” to improve motor function and secured with 9-0 non-absorbable epineurial sutures and fibrin glue (**d**). Reproduced by permission for Plastic and Reconstructive Surgery Global Open under the terms of the Creative Commons Attribution-Non Commercial-No Derivatives License 4.0 (CCBY-NC-ND) [146].

In cases of extensive ulnar nerve affection with atrophy of the distal muscles and clawing, a nerve transfer may be considered, i.e., an anterior interosseous nerve (AIN) transfer as a “supercharge” (Figure 7) [142,143,144,145,146]. The AIN transfer involves exploring the motor component of the ulnar nerve at the forearm level, followed by mobilizing the anterior interosseous nerve at the proximal part of the pronator quadratus muscle. The anterior interosseous nerve is then divided and connected end-to-side (in some cases, end-to-end) to the motor ulnar nerve branch (Figure 6). This procedure allows for the contribution of motor axons (supercharge) from the anterior interosseous nerve without any risk of decreasing pronation, as long as the pronator teres muscle is intact. This initiates regeneration and can lead to better functional recovery. The technique should probably only be reserved for specific severe cases, but it has shown promising results [142,143,144,145,146,147,148,149,150]. However, proper prospective randomized control trials with well-defined patients with severe ulnar nerve entrapment are required before any general recommendation can be made. 

### 5.2. Outcome of Surgery

The results of surgery to treat ulnar nerve entrapment at the elbow level vary between studies and evaluation methods. There is no general agreement on how the outcome of any treatment of patients with ulnar nerve entrapment should be followed, but patient-reported outcome measurements (PROMS), like PRUNE, are often used. Generally, only 60–85% of patients typically experience improvement or a cure of the symptoms and clinical signs of ulnar nerve entrapment. There is a risk of complications and worsening symptoms [2,40,42,82,83,121,122,123,133,151]. Predictors for poor outcomes after surgery are not clear, but the structural condition of the nerve and the presence of atrophy are important biological factors. Electrophysiological examination often shows metabolic conduction blocks with signs of axonal degeneration, which can result in less satisfied patients since nerve regeneration may be limited [23,152]. Transpositions carry a higher risk of complications, especially in patients who smoke or have diabetes [40,73]. Postoperative infections occur in approximately 3% of patients, while complex regional pain syndrome (CRPS), reduced sensation in the operation area, and neurogenic pain are direct nerve-related complications observed in 2%, 7%, and 8% of surgically treated patients, respectively [40].

### 5.3. Recurrence of Ulnar Nerve Symptoms and Revision Surgery

Symptoms may not improve or return after surgery, which is why a detailed medical history and clinical examination should be conducted to rule out any other causes besides direct nerve entrapment at the elbow level. In cases where the nerve is painfully dislocated, it can be treated through one of the described nerve transposition procedures [73,153,154,155]. A higher risk for revision surgery includes patients with workers’ compensation insurance, previous simultaneous bilateral surgery, a submuscular transposition as a primary procedure, spinal cervical disc herniation, a higher BMI, smoking and alcohol consumption, and pain problems after primary surgery, while the risk is lower among those with a higher age and a concomitant carpal tunnel release [153,156,157]. Revision surgery may result in complete or partial recovery, but residual problems, such as paresthesia, are the most frequent symptom, and patients with no improvement are common [155,158]. Again, there is a lack of clinical studies with patients suffering from recurrent or persistent ulnar nerve entrapment, despite a recent meta-analysis of the subject indicating that submuscular ulnar nerve transposition is superior to other procedures with respect to improvement of sensory and motor functions [71]. Still, general recommendations cannot be made regarding diagnostic criteria, indication for surgery, or selection of surgical method in persistent and recurrent ulnar nerve entrapment, although pain and sensory dysfunction seem to be prominent symptoms among such patients [71]. Thereby, it is difficult to generally judge the outcome of revision surgery for ulnar nerve entrapment, and further research is needed [137,159]. An interesting observation is that some patients, maybe based on the genetic constitution, tend to develop scarring around, or tethering of, nerves, which is defined as a variant of neuroma [160,161]. This may cause substantial remaining pain problems despite surgery [162]. Some patients also have an initial improvement after surgery but, with time, regain symptoms. There is a need for further research on the outcome of revision surgery with a sufficient follow-up time and definition of primary surgery, peroperative findings, type of secondary surgery, and patient characteristics, probably also involving analysis of proteomics and genomics of the patients. In contrast, if there is suspicion of another origin for the symptoms, further examination may be necessary to identify specific conditions. This may include an MRI, high-resolution ultrasound, electrophysiological examination, and suitable examination of any other neuropathy, including biomarkers, with the assistance of a neurologist [163,164].

## 6. Ulnar Tunnel Syndrome

### Ulnar Nerve Entrapment at Guyon’s Canal

Entrapment of the ulnar nerve at the wrist level is relatively uncommon [34,165]. External trauma or pressure against the nerve can be the cause. A thorough medical history and clinical examination are necessary to confirm the exact location of nerve compression. Clawing is a common symptom that occurs more frequently in ulnar tunnel syndrome than in ulnar nerve entrapment at the elbow. If necessary, additional examinations, such as MRI investigations, CT scans, and neurophysiology/electrophysiology tests, can be conducted, but these evaluations should not delay treatment. Ganglia is a common occurrence in this region and may result in only loss of motor function depending on its localization, which requires immediate surgery to prevent permanent nerve damage [166]. Entrapment may occur due to many various reasons, such as anatomical variations, synovial cysts, different types of tumors, and external compression, including ulnar artery thrombosis. In some cases, the problems may have a clear traumatic origin, such as localized pressure during bicycling and other activities. Conservative treatment may be successful in such cases. Surgical treatment usually involves simple decompression with division of the Guyon’s canal, but the type of procedure completely depends on the reason for the nerve affection. Division of the canal may be carried out in connection with carpal tunnel release, although expansion of the Guyon´s canal may occur in connection with a carpal tunnel release with improvement in two-point discrimination of also ulnar nerve innervated fingers [167]. The postoperative handling is the same for both surgical procedures [168,169].

## 7. Conclusions

Symptoms from ulnar nerve entrapment could be diagnostically difficult to interpret, including pain problems, which may be a prominent feature of the condition. The decision to perform surgery is often challenging due to the unpredictable outcome. From an international perspective, a lack of consensus concerning diagnostic criteria, indications for surgery, selection of surgical method, and how to evaluate patients with ulnar nerve entrapment. Conservative treatment could be successful in mild cases. When considering surgery, it is important to operate on a patient with an appropriate diagnosis and with an atraumatic surgical technique to prevent any scarring or tethering of the nerve. Preoperatively, advice to stop smoking, reviewing the ergonomics at the workplace and at home, and advice on a healthy lifestyle are crucial. A simple surgical procedure, such as simple decompression, should be used as the primary procedure. Recurrent problems can be treated with good results but require careful individual consideration of the right surgical treatment, which may include nerve transposition, as well as excluding other causes of the symptoms. Pre- and postoperative care is important. Unclear cases as well as cases with severe atrophy, where an AIN transfer is considered, should be referred to a special unit. The rich medical literature about ulnar nerve entrapment, without any clear guidelines from prospective and randomized as well as control studies, indicates that diagnostic and treatment-related problems occur, which is why it is important to perform multicenter studies to clarify indications for surgery, appropriate evaluation methods, and selection of surgical procedures for primary and recurrent ulnar nerve entrapment. It is crucial to recognize and manage sleeping disturbances and emotional symptoms early on in patients with severe neuropathic pain, as they can have a significant impact on the individual and add to their already heavy burden. Healthcare personnel should adopt a bio-psychosocial approach when treating patients to ensure comprehensive care.

## Figures and Tables

**Table 1:** A simple overview of variety of factors that may contribute to ulnar nerve entrapment at the elbow divided by different categories (for details see text).

Anatomical Etiologies and Trauma	Patient Characteristics	Concomitant Diseases	Occupational Factors	Socioeconomic Factors
Local anatomy at the elbow (ulnar groove, subluxation etc.)	Women (?)	Diabetes and other diseases (e.g., vitamin B12, folate deficiency) causing neuropathy	Work with vibrating hand-held tools	Low education level
Anomalous muscles (anconeus-epitrochlearis muscle) or displaced triceps muscle belly	Obesity	Carpal tunnel syndrome	Manual work—dependent on occupation	Low earnings
Elbow trauma (e.g., fractures) and subsequent osteosynthesis	Dysregulation of blood lipids	Inflammatory diseases at elbow (rheumatoid arthritis etc.)	Repetitive work tasks (e.g., extension-flexion movements)	High social assistance dependency
Heterotopic ossification	Smoking	Osteoarthritis	Holding a tool	Unemployment
Soft tissue masses (lipomas etc.)	Sleeping position	Extra- and intraneural cysts		Workers’ compensation
Tumours in brachial plexus and at proximal locations	Contralateral ulnar nerve entrapment	Spinal nerve root affection and cervical disc diseases (?)		
External pressure (e.g., leaning on elbow)		Neck and shoulder problems		
		Gout		

Specific etiologies or factors associated with ulnar tunnel syndrome are not included; the reader is referred to the text.

**Table 2:** Examples of techniques used to evaluate function and imaging of the ulnar nerve, surrounding tissues, and involved muscles in ulnar nerve entrapment.

Technique	Expected Findings	Comment
Conventional X-ray or Computerized Tomography (CT) of elbow Conventional X-ray or CT of lung	Skeletal abnormalities Skeletal tumours Ulnar nerve groove Lung pathology (e.g., tumour)	Skeletal parts evaluated 3-D reconstruction possible Fast examination before detailed biopsies etc.
Electrophysiology (neurography and electromyography)	Motor conduction velocity (MCV) Sensory conduction velocity (SCV) Orthodromic distal sensory nerve action potential (SNAP) Amplitude of compound motor action potential Inching technique	Possible simple grading based on neurography: - normal findings - impaired nerve conduction velocity - metabolic conduction block - signs of axonal degeneration
Ultrasound	Distal and proximal cross-sectional areas (CSAs) Longitudinal evaluation of antero-superior diameters (ASD) Neural echostructure Morphology of the epineurium Degree of nerve displacement affected by elbow flexion	Normative values available for CSA
Magnetic Resonance Imaging (MRI)	Signal intensity and size of nerve (CSA) Intra- and extraneural pathology (i.e., space-occupying lesions) Subluxation Muscle denervation	Non-invasive adjunct For example: (a) normal nerve = isointense compared to surrounding on T1 and T2-weighted images. (b) abnormal nerve = hyperintense on T2-images [short-tau inversion recovery (STIR) images]. (c) diffusion-weighted imaging (DWI) = additional technique to visualize ulnar nerve (mobility of water molecules ≥ increased mobility and altered water compartmentalization in nerve entrapment).

## Data Availability

Not applicable.
